# Clarifying the role of metformin and statistical interpretation in sleep quality research among patients with diabetes

**DOI:** 10.1097/MS9.0000000000003954

**Published:** 2025-09-23

**Authors:** Haseeb Safdar Ali, Esha Bilal, Muddassir Khalid, Mohammed Hammad Jaber Amin

**Affiliations:** a Department of Medicine, Nishtar Medical University, Multan, Pakistan; b Department of Medicine, Alzaiem Alazhari University, Khartoum, Sudan

**Keywords:** diabetes, metformin, sleep quality

*Dear Editor*,

We found the recent cross-sectional study by Hamayal *et al* and colleagues on the sleep quality of Pakistani patients with diabetes to be very interesting[1]. The high rate of poor sleep quality (82%) in this group shows how important the topic is in medicine. The authors deserve praise for bringing attention to a complication of diabetes that is often overlooked. However, after a thorough review, we found a number of methodological and interpretive issues that could affect the study’s conclusions. First, the manuscript underscores metformin use as a substantial factor in poor sleep, as indicated by Table 3 (86.4% poor sleepers; *P* = 0.002). However, this result comes from a bivariate chi-square analysis of subgroup distributions, which can’t show that there is an independent relationship. In the multivariable logistic regression (Table 5), metformin did not remain a significant predictor, while uncontrolled diabetes and comorbidities did. We need to clear up this contradiction. Putting too much weight on the unadjusted finding could make the role of metformin seem bigger than it is. Indeed, extensive adjusted analyses, exemplified by a UK Biobank study involving over 11 000 patients, revealed no independent correlation between metformin usage and sleep disturbances when accounting for age, HbA1c levels, and disease duration. In contrast, alternative non-metformin therapies exhibited more pronounced associations with sleep difficulties[2]. These results indicate that the emphasis should be on glycemic control and comorbidities, rather than metformin itself.

The authors also say that the model “correctly classified 74.3% of cases.” But since 82% of people don’t sleep well, a naive classifier that calls all patients “poor sleepers” would already be more accurate. This means that the reported classification rate doesn’t give useful information and could even be wrong. Current statistical advice says that logistic regression performance should be judged by things like the area under the receiver operating characteristic curve, calibration, and confusion matrices, not just how accurate the classification is^[3,4]^. Reporting these metrics would give a better idea of how useful the model is. In addition, a few minor inconsistencies warrant attention. The “None” medication category in Table 1 shows 30 (8.2%), but when rounded to one decimal place, 30/372 equals 8.1%. Figure 1 shows what is not included for “sleep disturbances prior to diabetes,” and the Methods section says that “any mental disorder prior to diabetes” is not included. It would be more open if this difference were explained for a better understanding of the study.

In conclusion, this study looks at a clinically important issue that has big effects on public health. The purpose of our comments is to improve the interpretation and point out places where things could be made clearer. To make the manuscript stronger and make sure that readers get the most accurate message that poor glycemic control and comorbidities, not metformin use itself, are the main causes of poor sleep in this population, there should be a reconciliation of bivariate and multivariate findings, the use of appropriate regression performance metrics, and minor corrections to reporting.

TITAN Guidelines: This manuscript complies with TITAN Guidelines, 2025, declaring no use of artificial intelligence[5].

## Data Availability

Not applicable.

## Research registration unique identifying number (UIN)

Not applicable for this letter to the editor.

## Peer and provenance statement

Not commissioned, externally peer-reviewed.
